# A more responsive, multi‐pronged strategy is needed to strengthen HIV healthcare for men who have sex with men in a decentralized health system: qualitative insights of a case study in the Kenyan coast

**DOI:** 10.1002/jia2.25597

**Published:** 2020-10-01

**Authors:** Elise M van der Elst, Rita Mudza, Justus M Onguso, Leonard Kiirika, Bernadette Kombo, Nassim Jahangir, Susan M Graham, Don Operario, Eduard J Sanders

**Affiliations:** ^1^ Kenya Medical Research Institute‐Wellcome Trust Research Programme Kilifi Kenya; ^2^ Department of Global Health Academic Medical Centre University of Amsterdam the Netherlands; ^3^ Jomo Kenyatta University of Agriculture and Technology Nairobi Kenya; ^4^ Institute for Biotechnology Research Jomo Kenyatta University of Agriculture and Technology Nairobi Kenya; ^5^ Department of Horticulture and Food Security School of Agriculture and Environmental Sciences Jomo Kenyatta University of Agriculture and Technology Nairobi Kenya; ^6^ Ministry of Health Kilifi County Kenya; ^7^ Departments of Medicine Global Health, and Epidemiology University of Washington Seattle WA USA; ^8^ Department of Behavioral and Social Sciences School of Public Health Brown University Providence RI USA; ^9^ Nuffield Department of Medicine University of Oxford Oxford United Kingdom

**Keywords:** HIV healthcare services, MSM, Kenya, decentralization, health equity

## Abstract

**Introduction:**

HIV healthcare services for men who have sex with men (MSM) in Kenya have not been openly provided because of persistent stigma and lack of healthcare capacity within Kenya’s decentralized health sector. Building on an evaluation of a developed online MSM sensitivity training programme offered to East and South African healthcare providers, this study assessed views and responses to strengthen HIV healthcare services for MSM in Kenya.

**Methods:**

The study was conducted between January and July 2017 in Kilifi County, coastal Kenya. Seventeen policymakers participated in an in‐depth interview and 59 stakeholders, who were purposively selected from three key groups (i.e. healthcare providers, implementing partners and members of MSM‐led community‐based organizations) took part in eight focus group discussions. Discussions aimed to understand gaps in service provision to MSM from different perspectives, to identify potential misconceptions, and to explore opportunities to improve MSM HIV healthcare services. Interviews and focus group discussions were recorded, transcribed verbatim and analysed using Braun and Clarke’s thematic analysis.

**Results:**

Participants’ responses revealed that all key groups navigated diverse challenges related to MSM HIV health services. Specific challenges included priority‐setting by county government staff; preparedness of leadership and management on MSM HIV issues at the facility level; data reporting at the implementation level and advocacy for MSM health equity. Strong power inequities were observed between policy leadership, healthcare providers and MSM, with MSM feeling blamed for their sexual orientation. MSM agency, as expressed in their actions to access HIV services, was significantly constrained by county context, but can potentially be improved by political will, professional support and a human rights approach.

**Conclusions:**

To strengthen HIV healthcare for MSM within a decentralized Kenyan health system, a more responsive, multi‐pronged strategy adaptable and relevant to MSM’s healthcare needs is required. Continued engagement with policy leadership, collaboration with health facilities, and partnerships with different community stakeholders are critical to improve HIV healthcare services for MSM.

## 1 INTRODUCTION

Kenya has among the largest HIV epidemics in the world with an estimated 1.6 million people living with HIV, 46,000 new infections, and 25,000 AIDS‐related deaths in 2018 [1], affecting both general and key populations (KP), including men who have sex with men (MSM). MSM have been engaged for longitudinal research in coastal Kenya since 2005 [2], and Sanders *et al*. [3] showed that MSM had an HIV‐incidence of 5.1 (95% CI: 2.6 to 9.8) per 100 person years in 2013. HIV incidence, however, has remained high in coastal Kenya despite programmatic pre‐exposure prophylaxis (PrEP) offered to MSM since 2017 [4]. While HIV transmission patterns within and between risk groups in Kenya are not well understood, recent HIV phylogeny research showed that 85% of transmission clusters was within risk groups, whereas 15% was shared between risk groups [5], suggesting that the HIV epidemic among MSM require targeted interventions.

Efforts by Kenya’s National AIDS Control Council (NACC) [6] to strengthen HIV healthcare services for MSM in Kenya [7, 8] have been obstructed by historical practices, policies criminalizing consensual same‐sex sexual practices [9], and deep‐rooted prejudice and patterns of stigmatization against male‐same‐sex practices [10, 11]. The use of a human rights based‐approach as a strategy to facilitate access to HIV prevention and care services by MSM vis‐à‐vis illegality of same‐sex sexual practices has caused profound confusion among policymakers, healthcare providers, implementing partners and MSM themselves [12]. This is a challenge for many African countries that simultaneously criminalize same‐sex practices while including sexual and gender minorities in national HIV health policy plans.

In 2013, responsibilities for overseeing the implementation of healthcare services were officially transferred from the national Government of Kenya and Ministry of Health (MoH) to local county governments [13, 14]. At the national level, the MoH, and the National AIDS and sexually transmitted infections (STI) Control Programme (NASCOP) retained their function formulating policies, guidelines, regulations and standards, while provision of technical guidance and support to healthcare staff was transferred to the counties [15]. Kenya comprises 47 counties, and it has been challenging to design, implement and assess technical changes in HIV prevention and care services [16], without a deeper understanding of the country’s complex political context [17]. Based on an analysis of the literature on priority setting, Barasa *et al*. [18] suggested that county governments should develop legislation that gives hospitals greater control over resources and key management functions. Similarly, in a systematic review of research on experiences of decentralization in sub‐Saharan Africa, Zon and others [19] underscored the complexity of implementing decentralization schemes, and the need to transfer and increase general administrative capacities and resources before introducing more complex functions. Bossert *et al*. [20] also discussed issues of decision space in the context of financing and resources allocation in Ghana, Zambia, and Uganda, and showed how coordination and monitoring mechanisms among the stakeholders might be a challenge with decentralization implementation bodies [19, 21, 22, 23]. While these studies did not assess services for vulnerable populations at decentralized levels, Makofane argued that MSM are inadequately addressed in Africa’s AIDS National Strategic Programming [23]. The objective of this paper is to gain a better understanding of Kenya’s evolving awareness of MSM inclusiveness in health services planning, using Kilifi County as a case study. Kilifi is a compelling context for analysis because of its relatively large MSM population and previous work by Kilifi‐based researchers who have engaged MSM as early as 2005 [24, 25]. One particularly relevant Kilifi‐based research study involved a NASCOP‐endorsed MSM online training designed to strengthen healthcare providers’ skills to support healthcare services for MSM patients in East and South Africa, that was evaluated in Kilifi County [10]. This study served as a starting point in the discussions with the different stakeholders on what was needed to improve HIV service delivery for MSM to establish synergies, and to create a collaborative platform to further strengthen HIV healthcare services for MSM.

## 2 METHODS

The study was conducted between January and July 2017 in and around the three largest government health facilities (Kilifi County Hospital in Kilifi, Malindi sub‐County Hospital in Malindi, and Mtwapa Health Center in Mtwapa) that serve approximately 1.4 million people over an area of 12,246 km^2^. Since 2005, the Kenya Medical Research Institute‐Wellcome Trust Research (KWTRP) Programme has offered HIV prevention and care services for MSM at a stand‐alone clinic in Mtwapa, and at the sub‐County Hospital in Malindi and the programme campus in Kilifi since 2008. The Kilifi County health offices are located adjacent to the campus of the KWTRP. Through in‐depth interviews (IDI) and focus group discussions (FGD), we collected narrative data of participants’ experiences and perceptions regarding the provision and quality of HIV healthcare services for MSM in Kilifi County.

### 2.1 Participants and procedures

A total of 17 key informants (KI) were purposively selected to participate in IDI, including four staff from the MoH, two members from the County Health Management Team, two members from sub‐County Health Management Team, three facility in‐chargers, and six implementing partners of Kilifi County’s main Non‐Governmental Organizations (NGOs). The implementing partners were either commissioned by Kilifi County’s MoH, NACC, or NASCOP to coordinate training and education, and implemented complementary comprehensive HIV services. KI thoughts and hypotheses on HIV and access to HIV healthcare for MSM were used to finalize semi‐structured focus group topic guides.

Participants for FGDs were selectively sampled and organized by two main shared characteristics: residential location in Kilifi County and knowledge or familiarity with MSM through professional, community, or personal experience, yielding discrete focus group samples (i.e. HIV healthcare professionals, implementing stakeholders and members of a community‐based organization in support of MSM health and human rights). Three FGDs were conducted with HIV healthcare providers (N = 23) from healthcare facilities, two FGDs were held with people working or volunteering for NGO (N = 14) (each FGD including seven to eight participants), and three FGDs were held with members of Kilifi County’s main MSM‐led Community‐Based Organizations (CBO) (N = 22) across Malindi, Kilifi and Mtwapa (each FGD including six to nine participants). Participants were informed that the purpose of FGDs was to discuss barriers and facilitators to accessing HIV services, and to share recommendations to improving MSM HIV prevention and care services in Kilifi County. In a final feedback FGD, preliminary study findings were presented for validation to eight representatives of the above‐mentioned groups of FGD participants.

Semi‐structured IDI topic guides were used to collect detailed data on current interventions for MSM, MSM guidelines, county support, and reporting tools. Semi‐structured FGD topic guides focused on perceptions and experiences of local healthcare delivery for MSM including healthcare availability, quality and accessibility. IDIs lasted approximately 60 minutes while FGDs took up to 90 minutes. All IDIs and FGDs were audio‐recorded, and socio‐demographic characteristics of each participant were obtained in a brief questionnaire. A member of the socio‐behavioural research team, fluent in English, Swahili and the local native language “Mijikenda,” conducted the IDIs and moderated the FGDs. Consents and topic guides were translated by the HIV project’s studies coordinator at KEMRI, who has extensive experience with HIV studies.

### 2.2 Ethical considerations

Participants were informed about the study aims and provided signed informed consent. The study procedures were approved by the ethical review board at KEMRI and the Kilifi County Department of Health Research Committee (KEMRI/Scientific and Ethics Review Unit Ref: KEMRI/SERU/CGMR‐C/061/3372). Participants received 500 Kenyan shillings (approximately US$6) for travel and out of pocket expenses. Reimbursement amounts were determined based on previous studies with these groups and were deemed appropriate and non‐coercive according to local standards [26].

### 2.3 Analysis

The audio files for the IDIs and FGDs were transcribed verbatim, and (if applicable) translated by a socio‐science qualitative researcher with linguistic competency in Swahili, English and Mijikenda. The transcripts were uploaded in NVivo 11 software for data management. Analyses followed Braun and Clarke’s thematic approach for qualitative data [27], which involved systematic coding, identifying and defining concepts emerging from the data across the data set, mapping the concepts, creating typologies, finding associations between concepts and seeking explanations from the data. Findings were triangulated across sites and between respondents.

## 3 RESULTS

Table 1 provides summary characteristics of the study participants. A total of 76 participants took part in the study: 17 participants in the IDIs and 59 participants in one of eight FGDs. Of the 17 IDI participants, 14 interviewees derived from the MoH, including 11 county representatives of NASCOP and three supervisors. Women and men were equally represented in the IDIs, their median age was 42 years (range: 34 to 49), they had an average of nine years HIV experience, and 29% was Muslim. In the three healthcare providers’ FGDs, a total of 23 participated, eight from Malindi, eight from Kilifi, and seven from Mtwapa. Irrespective of their clinical job role (clinicians, nurses, or HIV counselors), all had received previous training on how to counsel MSM clients, and they had an average of six years of experience in the HIV field. The median age was 36 years (range 27 to 46) and the majority (70%) were women. Twenty‐two healthcare providers were Christian, and one healthcare provider was Muslim. In the two NGO FGDs, 14 implementing partners took part, their median age was 33 years (range: 26 to 40), men were 57% and they had an average of eight years of experience in the HIV field. For the three FGDs held with 22 MSM, participants were purposefully selected for their knowledge of healthcare provided to MSM in Kilifi County. The median age of the MSM participants was 27 years (range: 18 to 35), and 50% was Muslim.

**Table 1:** Socio‐demographic characteristics of participants in Kilifi case study, 2017

Characteristic	In‐depth interviews (N)	Focus group discussions (N)	Focus group discussions (N)N	Focus group discussions (N)
Number of participants	17	23	14	22
Specialty area	Health policymakers	HIV healthcare providers	Leaders of NGO	Leaders of MSM CBO
Women	9	16	6	0
Men	8	7	8	22
Median age (range)	41.5 (34 to 49)	36 (27 to 45)	33 (26 to 40)	26.5 (18 to 35)
Religion				
Christian	12	22	7	9
Muslim	5	1	7	11
Other				2
Education attainment				
Primary education		1		11
Secondary education	6	5	4	6
Diploma	11	15	8	2
Degree		2	2	3
Years working in HIV field				
1 to 5	6	15	–	–
>5	11	8	–	–

CBO, Community Based Organization; NGO, Non‐Governmental Organization.

Irrespective of participants’ characteristics, responses revealed that all key groups navigated diverse challenges related to MSM HIV health services. Specific challenges included priority‐setting by county government staff; preparedness of leadership and management on MSM HIV issues at the facility level; data reporting at the implementation level; and advocacy for MSM health. Strong power inequities were observed between policy leadership, healthcare providers and MSM, with MSM feeling blamed for their sexual orientation. MSM agency, as expressed in their ability to access HIV services, was significantly constrained by county context.

### 3.1 Priority‐setting by county government

Six years into decentralization, Kilifi County has not given as much priority to HIV programming as compared to other major health concerns such as malaria, tuberculosis, diarrhoea and other non‐communicable diseases. Respondents recognized that HIV, and particularly same‐sex sexuality, are still extremely sensitive topics. However, all strongly advocated for MSM inclusion and felt that a breakthrough was imminent – that is having MSM patients openly to be attended to in public facilities. KIs associated the Kenyan president’s statement on same‐sex sexuality with the local county’s priority‐setting and decision making:

*“The county government has not yet taken it [MSM HIV healthcare] as a responsibility. I think it is just revolving the fact that it is not accepted and holistically we saw the president not accepting it, so they [county staff] are like who are we to do what other people are rejecting, why should we pretend to…”*

The same respondent continued:

*“… HIV healthcare is in NASCOP’s guidelines and is the reason why non‐governmental organizations and funders are taking it up. So, it is not like it is not possible, it is very possible, it’s just that structures have to be re‐established again to have it fit the health community in a way that healthcare for MSM can be provided to and received by MSM without fear at the facility level. We [policy makers] are the liaison, and it is our goal to eradicate HIV, so we need to work with partners, organizations, the community, and most importantly we need to involve the MSM on all the reasons why we still have high HIV infections…”. (IDI/policy maker/F)*

This quote also suggests that much of the effort to include MSM into the HIV prevention and care continuum is done by implementing partners through bilateral donors, rather than by the county itself. Financial prioritization was highlighted as the precursor of all quality care within the Comprehensive Care Centre (CCC) and was especially crucial for directing services to MSM who are living with HIV who otherwise would lack access to healthcare. Due to the influences of NASCOP and implementing partners, a more positive trend on budgeting, planning and prioritization of county government’s HIV prevention and care programme, catering for MSM‐specific needs for tailored services, was noted:

*“If you look at our budget, HIV has always received zero support from the county government not only for key populations, but for the whole HIV management programme…yeah, zero finances…, all along we have been relying on NASCOP and depending on partners. HIV is still looked upon at as a programme belonging to NASCOP.., we need to have a clear memorandum of understanding between the partners and the county government to make sure that the finances are there to continue HIV services especially for MSM living with HIV, to reduce stigma and discrimination…”. (IDI/policy maker/M)*

### 3.2 Preparedness of leadership and management at the facility level

The rush towards decentralization combined with limited technical capacity and guidance at the local level further de‐prioritized the already stigmatized HIV health services in Kilifi. Due to the dynamics of stigma, manifested in healthcare managers’ disapproval of same‐sex‐sexuality practices and general societal disregard for MSM community members, healthcare providers commonly refused MSM healthcare services to MSM clients:

*“… healthcare workers don’t feel that they have support from let’s say their managers or their in‐charges when it comes to attending to MSM. For example, people working in the CCC are assumed to be positive, those people working with MSM are assumed to also be MSM… Even the manager or in‐charge, when he reaches “the camp” [CCC], he peeps and says: “Now this place, ‘ai’, me I can’t go in”. “Does that really support?” (FGD/HCP/F)*

In contrast, MSM described the need for HIV services where they could safely disclose their identity and freely talk about specific sexual health issues, and recommended skills training to improve capacities among healthcare providers:

*“At XX, the doctors were shocked to learn about my [same‐sex] sexuality. They [healthcare providers] discussed it with colleagues in tones that could be heard by other patients. You know, it will take time for them [healthcare providers] to accept the sexual aspects of MSM, they need sensitization, as we need education and to know our rights.” (FGD/MSM)*

When the strategic planning was done at the county level, policymakers were already sceptical about the implementation of MSM HIV healthcare services. Reasons for their scepticism ranged from the county’s overstretched healthcare system and related organizational and staffing issues including: the structure of leadership and management [including the county‐wide strikes of clinical officers and nurses at the time]; lack of ownership; and experiencing “teething” problems by new healthcare staff. Across sites, and between respondents, limited resources were identified as reasons for underperforming country‐level responses, ranging from staff shortages, lack of STI diagnostic testing capacity and shortage of medications for treatment. NGO leaders stressed the importance of endorsing MSM policy reforms and the integration of MSM guidelines to facilitate appropriate services for MSM. Drawing on respondents’ different reflections on what needs to be done to improve MSM inclusiveness in health services, one of the policymakers elucidated:

*“I think what needs to be done is to create a very strong network to be adapted for MSM health so that whatever improvements in one area should be actually known to the other party, there should… has to be a lot of transparency in all these activities, there is supposed to be a lot of cooperation and coordination, because we are the custodians of health…”. (IDI/policy maker/M)*

### 3.3 Data reporting, impacting services implementation

Implementing partners described inaccurate reports as a harmful negligence, leading to misinformation and altering decision making. They felt that improving accurate data would be crucial to strengthening MSM HIV healthcare services. For example the recently revised reporting tool (MoH form 731), used to prepare monthly reports on CCC’s patients and feeding into Kenya’s District Health Information System, does not capture the different KP categories, such as MSM. MSM status is only documented at the HIV testing and counselling register, different from the MoH 731 tool‐ and is subsequently lost at the national level. The following statement represents responses from various participants:

*“We have data, but it is not specifically saying who the HIV patients are. Only, when we go back to the HTC [HIV Testing and Counselling] register there is a place specifically indicating whether so and so is MSM, a sex worker, or an IDU. But basically, it ends here, at the HTC register”. (FGD/NGO/F)*

Implementing partners stressed that with proper data collection tools would allow them to contribute more efficiently towards appropriate planning and implementation of HIV prevention and care programmes that target MSM.

### 3.4 Advocacy of MSM health

All participants pointed out that in order to give momentum to and endorse policy reforms that support MSM HIV services, members from the MSM communities need to be acknowledged. As for now MSM issues are silent issues in the communities and in healthcare facilities.

MSM respondents expressed the dire need for internal coherence and consistency. They urged the different policymakers and partners to address the structural inequities MSM face both at county and national levels. Following an exchange about their experiences at public health facilities, one MSM participant summarized their discussion, representing the point of view of his fellow MSM participants:

*“Many of us [MSM] experience trauma due to prejudice and discrimination. We are not welcome at health facilities, and many of us discontinue care, yet we are in need of critical HIV services …”. (FGD/MSM)*.

They strongly recommended improving the clinical training of HIV healthcare providers on matters specific to MSM issues, as well as strengthening collaboration between MSM health advocacy groups together with national entities such as NACC and NASCOP. The highest priority expressed by MSM respondents was the need to raise awareness of MSM health equity in Kenya’s HIV prevention and care continuum.

## 4 DISCUSSION

This case study focused on the necessary conditions required to bring about collectively organized responses to strengthen HIV healthcare services delivery for MSM in a relatively young decentralized health system. Ideally, accurate data on MSM size estimates would have been instrumental for appropriate programming. The absence of it, however, made the case study (conducted in 2017) more valuable, that is the stakeholders’ perspectives are relevant as to MSM’s current situation. In accordance with the latest Kenya AIDS Response Progress Report [28, 29], the study showed challenges with quality and coverage of public HIV healthcare with regard to infrastructure and workforce. Concerns about MSM‐specific needs, such as confidentiality, MSM‐specific services, public stigmatization and poor or no linkage to national reporting systems, restricted integration of MSM into the existing county health system. Efforts to improve the attitudes of relevant health policy agents (e.g. staff, managers and other policymakers) with regard to the basic dignity and rights to inclusive health services can contribute to MSM’s trust in the health system at the local level [30]. Here, Rondinelli’s early appraisal of East African decentralization programmes [31], including characterization of limited resources, infrastructure, workforce and commodities management in general, were still applicable. Also, in line with previous findings [28], the managers or “in‐chargers,” lacked trust in the health system which outwardly contributed to fear of engaging MSM into prevention and care programmes across the local units. Although the number of relevant publications on decentralization and its impact on MSM health services in sub‐Saharan Africa is limited, our findings correspond with the critical need to complement institutional capacity and political and economic support with specific MSM programming [18]. For example as much as test‐and‐treat programmes in sub‐Saharan Africa have evinced general success [32], mobilizing MSM to engage with HIV testing has been challenging at the local level [33]. Given the empowering effects of decentralization on county‐level policymakers, prioritization of HIV services for MSM can only result in the presence of clear leadership commitment, managerial capacities, and provision of funding. Implementation of local HIV prevention and the care continuum for MSM must be introduced incrementally and accompanied by comprehensive guidelines on how to provide effective and sensitive MSM HIV services. In order to achieve improved MSM HIV healthcare services on a larger scale, reporting tools should aggregate data into transmission risk groups, including MSM, men who have sex with both men and women, men who receive payment for sex with cash, living expenses or goods and men who inject drugs, explicitly indicating the services needed at the local level as well as specifying performance criteria that should be met. It is of note that the focus of this study was on HIV healthcare services for MSM in decentralized public health facilities, however, other models of differentiated HIV services delivery for MSM could be considered and may be an alternative option to the current public HIV‐related clinics and patient–provider relations.

If decentralization structures would not have been created in Kenya, the HIV response would have remained highly centralized, local decision‐making powers would not have been consolidated, community involvement would have remained weak, capacity gaps continued, and the policy focus would have exclusively emphasized outcome (e.g. metrics) over process (e.g. engagement).

The impacts of decentralization with regard to provision of healthcare are experienced by all counties and by all populations. However, as MSM are uniquely vulnerable to substandard services, there is a need for stronger and focused commitment to the provision of prevention and care including antiretroviral drugs for treating and preventing HIV infection for their unique risks. Improvements in healthcare services for MSM cannot happen without endorsing policy reforms that support integration of MSM services unambiguously at the county level. Referring to Kenya’s guidelines [7, 34, 35], barriers to the right to necessary and appropriate health services should immediately be identified and addressed [36], and concrete recommendations for healthcare providers, policymakers and stakeholders should be invested in as strategies to assure the right to health for MSM populations. First, MSM rights should be incorporated into the country’s legal and cultural code, with a focus on laws and decriminalization of same‐sex behaviour in order to ensure that access to health services are not impacted. Second, the County Management should take a lead in norm diffusion, by implementing policies countering all forms of homophobia. Third, HIV‐related service delivery for MSM should be prioritized in order to sufficiently impact the HIV epidemic in the county. Fourth, substantial improvements are needed to translate NASCOP and NACC’s centralized guidelines to the local levels. Finally, decentralized governance, including leadership and training should assist with implementation of policies that exist at the central level for HIV prevention and care for MSM.

Limitations to this research must be acknowledged. This study took place in Kilifi County, where MSM research has occurred since 2005; therefore, findings might not be generalizable to other counties in Kenya. This paper focuses exclusively on MSM and does not take into account other KP or competing needs for resources. We recognize that participants in this sample may not have represented a broader MSM population in Kenya, as many MSM may remain hidden. Furthermore, participants might have been prone to socially desirable reporting about their attitudes and experiences related to health services for MSM. Finally, it should be acknowledged that we based the above conclusions on limited empirical evidence, recognizing that the use of qualitative methods produces inherently subjective data which may impose limits on the transferability of the knowledge uncovered.

## 5 CONCLUSIONS

To strengthen HIV healthcare for MSM within a decentralized Kenyan health system, a more responsive, multi‐pronged strategy adaptable and relevant to local county settings is needed. High‐level government decision makers need to acknowledge that HIV transmission occurs within marginalized groups and consolidate expert guidance to support implementation of differentiated HIV services delivery for MSM. Continued engagement with policy leadership, collaboration with health facilities, partnerships with different community stakeholders and most importantly the voices of the MSM communities being served are critical to improve MSM HIV healthcare services.

## COMPETING INTERESTS

The authors declare that they have no competing interests.

## AUTHORS’ CONTRIBUTIONS

EMvdE, RM, NJ, EG, LK, JO, SG, DO and EJS contributed significantly to the study design. EMvdE and EJS conceived the study. RM conducted the FGDs and interviews. EMvdE, RM and BK analysed the data. EMvdE, RM and EJS discussed full texts. EMvdE and RM drafted the manuscript. DO, SG and EJS critically edited the manuscript. All authors read and approved the manuscript.
